# Internet-based cognitive behavioural self-help for premenstrual syndrome: study protocol for a randomised controlled trial

**DOI:** 10.1186/1745-6215-15-472

**Published:** 2014-12-02

**Authors:** Johanna N Kues, Carolyn Janda, Maria Kleinstäuber, Cornelia Weise

**Affiliations:** Department of Psychology, Division of Clinical Psychology and Psychotherapy, Philipps-University of Marburg, Gutenbergstr. 18, 35032 Marburg, Germany; Department of Behavioural Sciences and Learning, Linnaeus Centre HEAD, Linköping University, Campus Valla, house I, 58183 Linköping, Sweden

**Keywords:** Premenstrual syndrome, Premenstrual dysphoric disorder, Internet-based cognitive behavioural therapy, Study protocol

## Abstract

**Background:**

With a prevalence of 3 to 8% among women of reproductive age, severe premenstrual symptoms are very common. Symptoms range from emotional and cognitive to physical changes. Severe symptoms (that is, premenstrual syndrome) can have a strong impact on everyday functioning and quality of life. Impairment can be as serious as that of dysthymic disorders. Many affected women receive either no treatment at all or are unsatisfied with their treatment. Although there is some evidence for the reduction of distress through cognitive behavioural therapy, there are only a small number of randomised controlled trials carefully investigating the efficacy of this psychotherapeutic approach. Thus, this study aims to evaluate the efficacy of a cognitive behavioural self-help treatment for women suffering from premenstrual syndrome.

**Methods/design:**

The study is conducted as a randomised controlled trial. The complex diagnostic assessment includes the completion of a symptom diary over two consecutive cycles and a telephone interview. Eligible women are randomly assigned to either a treatment or a wait-list control group. The intervention is based on cognitive behavioural therapy principles and is provided via the internet. It consists of 14 different modules on which participants work over 8 consecutive weeks. In addition to written information, participants receive email feedback from a clinical psychologist on a weekly basis. Participants assigned to the wait-list receive the treatment after the end of the waiting period (8 weeks). The primary outcome measure is the Premenstrual Syndrome Impairment Measure. Secondary outcomes include the Premenstrual Syndrome Coping Measure, the Short-Form Social Support Questionnaire, the Questionnaire for the Assessment of Relationship Quality, and the Perceived Stress Scale. Data is collected during the premenstrual (luteal) phase at pre-treatment, post-treatment, and 6-month follow-up.

**Discussion:**

So far, there is no study investigating internet-based cognitive behavioural therapy for premenstrual syndrome. The programme approaches the problem of high prevalence in combination with severe impairment and insufficient treatment options.

**Trial registration:**

ClinicalTrials.gov: NCT01961479, 9 October 2013.

## Background

A large proportion of women of reproductive age suffer from premenstrual symptoms. Some of the typical and frequently reported premenstrual complaints are physical discomfort, affect lability, anxiety, depressed mood, fatigability, hopelessness, and irritability [1]. The symptoms can lead to severe impairment at work, in social activities, or relationships [2]. The marital relationship is often impaired most intensely in comparison to other social relationships and activities outside the home [3, 4]. Quality of life is lowered to a degree comparable to that of dysthymic disorders [3] and suicidal tendency is increased [1].

According to the impairment caused by the symptoms, premenstrual syndrome (PMS) can be distinguished from a more severe form, the premenstrual dysphoric disorder (PMDD). PMDD is associated with a higher number, severity, duration, and quality of symptoms [5]. Consequently, PMS and PMDD can be arranged on a continuum. The central feature of both disorders is the cyclic pattern of symptoms: the symptoms arise during the final premenstrual phase and diminish with or a few days after the beginning of menses.

Only PMDD represents a clinical, diagnostic entity. In the *Diagnostic and Statistical Manual of Mental Disorders*, fourth edition text revision (DSM-IV-TR), PMDD was only included in the appendix at a research criterion stage [6]. Since DSM-5 [7] PMDD has been outlined as a distinct diagnostic category. (Wording of PMDD criteria in this text is simplified and abbreviated. For exact wording, please consult the DSM-5 manual [7]). To diagnose PMDD, at least five symptoms out of eleven (including at least one out of the first four affective symptoms) have to be confirmed by prospective daily self-ratings of PMS symptoms in the form of a diary over two consecutive menstrual cycles [7]. These symptoms include: (1) affective lability; (2) irritability or anger or increased interpersonal conflicts; (3) depressed mood; (4) anxiety or tension; (5) decreased interest; (6) difficulty in concentrating; (7) lethargy, fatigability, or lack of energy; (8) change in appetite; (9) hypersomnia or insomnia; (10) a sense of being overwhelmed or out of control; and (11) other physical symptoms (for example, breast tenderness, pain). The symptoms have to exist in the majority of cycles over the preceding 12 months and must cause significant distress or interference. Symptoms are not an exacerbation of the symptoms of another mental disorder; however, other mental disorders may co-occur. With the inclusion of PMDD in the DSM-5, some changes have been implemented [8]: "distress" in addition to interference, a more precise timing of the onset and the offset of the premenstrual symptoms, possibility of co-occurrence of other mental disorders, possibility of a provisional diagnosis based on clinical history, and distinctiveness from substance use or another medical condition. In particular, the possibility of a provisional diagnosis is an important change as it allows an earlier diagnosis and thus earlier access to healthcare for suffering women [9]. Also the extension of the interference criteria with the expression of distress is essential as it takes into account that women may maintain their function with a high level of distress without suffering from interference in functioning [10].

As already mentioned, PMS does not represent a distinct clinical entity and the distinction between PMS and PMDD remains unclear [11]. The American Congress of Obstetricians and Gynecologists (ACOG) [5] suggests some diagnostic criteria for PMS that require only one symptom out of a list of ten affective and somatic symptoms [5]. They seem to focus more on the impairment and distress than on a specified number of symptoms [3, 10].

The prevalence of PMS and PMDD differs not only due to differences in severity but also due to differences in methodology and diagnostic criteria [3]. About 75% of women of reproductive age report at least one premenstrual symptom [1]; 18% of women of reproductive age suffer from PMS [1]. Prevalence for PMDD based on the DSM-IV-TR ranges from 3 to 8% [10], with a 12-month prevalence of 5.3% [1].

About 85% of women with PMS are considerably impaired and seek treatment [12], but only 20% receive some kind of psychological treatment [1]. The direct costs for the healthcare system and especially the indirect costs through the loss of work productivity are striking [3, 13]: having PMS is associated with an annual increase of $59 in direct and up to $4,333 in indirect costs per patient [13].

Consequently, given the high prevalence and the negative impact on functioning, effective treatment is needed. Common treatments are pharmacotherapy (for example, antidepressants, anxiolytics, cycle-modifying hormonal agents), lifestyle changes (for example, exercise, dietary recommendations, relaxation therapy), complementary therapies such as nutritional supplements (for example, vitamin D, calcium, magnesium) and natural products (for example, St John’s wort extract, vitex agnus castus extract, evening primrose oil), and cognitive behavioural therapy (CBT) [14–16].

The Royal College of Obstetricians and Gynaecologists (RCOG) suggests a special treatment regimen for the management of severe PMS which is introduced in the following section [17]. Although complementary therapies have grown in popularity [18] there is no compelling evidence for their use [17, 19]. For lifestyle changes, sufficient randomised controlled trials (RCTs) are lacking as well [16, 17]. Nevertheless, some evidence exists for lifestyle changes (for example, exercise [20], dietary recommendations [21], relaxation therapy [22]) as well as for nutritional supplements (for example, vitamin D and calcium [23], magnesium [24]), and natural products (agnus castus extract [25]). Both complementary therapy (vitamin B6) and lifestyle changes (exercise) are recommended by the RCOG [17].

Common cycle-modifying hormonal agents that are used in the treatment of PMS/PMDD are the combined oral contraceptive pill, oestrogen, progesterone, and gonadotrophin-releasing hormone (GnRH). Evidence for the administration of progesterone [26] as well as for the use of second-generation combined oral contraceptives is lacking; in particular, RCTs are rare [14]. By contrast, the administration of oestrogen via oestradiol patches or implants [27] as well as the use of GnRH has proven effective [28]. However, the named hormonal therapies are associated with severe side effects [14, 29] which sometimes resemble or intensify premenstrual symptoms [14]. Regardless, the use of a new combined pill including progestin which has not been derived from testosterone (Yasmin®, Bayer Health Care, Leverkusen, Germany) is promising and its application is thus recommended by the RCOG [17].

Furthermore, selective serotonin re-uptake inhibitors (SSRIs) are recommended as one of the first-line pharmacological management options [17]. Four meta-analyses support the efficacy of SSRIs [30–33]. However, side effects of SSRI treatment [30, 34] lead to high rates of withdrawal from treatment. Further problems are the high placebo reaction in patients suffering from PMS [35] and the low responder rate of 40% [32].

Because of the problems of pharmacotherapy, CBT has been suggested as an additional treatment approach [36, 37] and has even been named a routine treatment option in the guidelines of the RCOG [17]. Theoretical substantiation for the adoption of CBT for PMS/PMDD comes from the cognitive model for PMS by Blake and colleagues [38]. The model emphasizes the importance of cognitive appraisal of the premenstrual symptoms and the associated distress. Here, CBT can intervene by questioning the attributions and by teaching coping skills. In addition, CBT has been shown to be effective for disorders sharing common symptoms with PMS/PMDD (for example, affective and somatic symptom disorder) [39]. Some studies showed promising results for CBT interventions for PMS/PMDD. In a meta-analysis examining CBT for PMS, nine studies were included [36]. The authors found medium effect sizes for reducing anxiety and depression. Another meta-analysis, containing 22 studies, revealed small to medium effects for CBT and serotonergic antidepressants concerning different outcomes (for example, mood and functional impairment) [40]. To date, only a small number of well designed RCTs have investigated the efficacy of CBT for PMS [36, 39]. Methodological limitations are, for instance, a lack of description of the randomization and treatment procedure, substantial loss to follow-up, and potential reporting bias. Besides, they show partially contradicting findings with unsatisfactory [40] or lacking evidence [39] contrasting findings of significant improvement [36]. Thus, one aim of the current study is to develop a well-designed, CBT-oriented self-help programme and to examine its efficacy for distress reduction in PMS patients.

The high percentage of women being dissatisfied with their actual treatment [41] reveals the importance of new approaches for treating PMS. Our treatment is conducted as an internet-based programme. This decision was reached for the following reasons. First, an internet-based approach enables us to provide treatment for a greater number of women and can therefore reduce waiting time for patients [42]. Second, stigma associated with seeing a psychologist and conveying sensitive information to a person can be reduced [42, 43]. This advantage seems to be particularly important because of taboos and stigmata linked with the topics of menstruation and PMS [44, 45], the women’s fear and experience of not being taken seriously [38, 41, 46], and their mainly medically oriented perception of PMS [38]. Positive effects of internet-based CBT (iCBT) in reducing impairment or symptom severity have been found for several functional syndromes or psychosomatic disorders (for example, pain, headache [47], or tinnitus [48]) as well as for depression [49]. Hence, it is highly probable that an iCBT programme can equally well target the somatic symptoms and the affective symptoms that go along with PMS/PMDD.

This study aims to evaluate the efficacy of an iCBT self-help programme for reducing mental and functional impairment in women suffering from severe PMS or from PMDD and improving their coping strategies. First, we hypothesize that the treatment group receiving the iCBT will show a lower impairment than the wait-list control group. Second, we hypothesize that the treatment group improves its coping strategies in comparison to the wait-list control group.

## Method

### Study design and population

This study will be implemented as an RCT including an experimental group and a wait-list control group. The study is approved by the Ethics Committee at the Department of Psychology of the Philipps University Marburg (2013–09) and was registered under clinicaltrials.gov (NCT01961479). Prior to participation in the research, informed consent was obtained.

Eligible women are between 18 and 45 years of age. They are required to have sufficient knowledge of German and access to the internet. Participants have to meet the DSM-5 criteria of PMDD [7] or the criteria of severe PMS as outlined in the guidelines of the ACOG [5].

Exclusion criteria for the study are as follows:

1. Diagnosis of a psychosis or a bipolar disorder
2. Diagnosis of an eating disorder
3. Diagnosis of moderate or severe depression
4. Diagnosis of somatic symptom disorder
5. Participation in psychotherapy due to premenstrual symptoms (currently or in the past)
6. Acute suicidal tendencies
7. Birth of a child or lactation during the last 3 months
8. Pregnancy
9. Gynaecological diseases (hysterectomy, oophorectomy, gynaecological cancer, polycystic ovary syndrome, endometriosis, infertility)
10. Begin or change in taking of antidepressants, benzodiazepines/antipsychotics, combined oral contraceptives, or hormones during the last 3 months

### Sample size and power calculations

Effect sizes of the difference between an iCBT programme for PMS and a wait-list control group cannot be estimated precisely because the combination of these two approaches – CBT and internet-based interventions – for treating PMS has not yet been evaluated systematically.

First, there is currently no internet-based self-help programme for PMS. However, iCBT has been found to be effective for disorders sharing common symptoms with PMDD (for example, affective and somatic disorders) [39]. Large effects for internet-based treatments with psychologist support for depression and anxiety disorders compared to control conditions were found in a meta-analysis [49]. The effects found for internet-delivered treatments aimed at health problems (pain and headache) were comparable to the effects for face-to-face interventions [47].

Second, CBT face-to-face programmes for PMS are not well evaluated and suffer from a limited methodological quality as well [36, 39]. In one meta-analysis examining CBT for PMS [36] the authors found medium effect sizes. Small to medium effect sizes were found in another meta-analysis comparing CBT and pharmacological interventions [40].

By considering the different effect sizes found in the cited studies and the insufficient current state of research, we based our sample size calculation on an expected medium effect size (*f* = 0.25, α = 0.05, power 80%). The validity and retest reliability of two applied tests is evaluated in a pilot study and thus is not known yet. Therefore, we assumed a conservative retest reliability. For a 2 × 2 multivariate analysis of variance (MANOVA) the total sample size is set to *N* = 128.

### Patient recruitment, randomization and procedure of the treatment

Participants are recruited via articles in newspapers, family doctors and gynaecologists, flyers, and different social networks.

**Figure 1:**
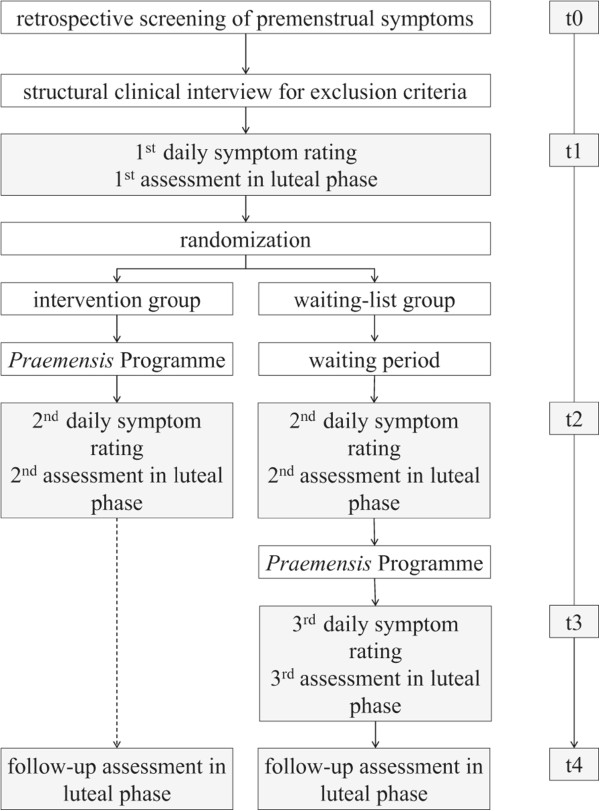
**Study process.** t = measurement points: t0 = screening for basic inclusion criteria. t1 = first assessment of primary and secondary outcome in luteal phase, first and second cycle of the symptom diary. t2 = second assessment in luteal phase, third and fourth cycle of the symptom diary, approximately 2 months after admission. t3 = for the wait-list group only, third assessment of primary and secondary outcome in luteal phase, fifth and sixth cycle of the symptom diary, approximately 6 months after admission. t4 = for the treatment group: third assessment of primary and secondary outcome in luteal phase, approximately 10 months after admission; for the wait-list group: fourth assessment of primary and secondary outcome in luteal phase, approximately 14 months after admission.

Eligible participants undergo four assessments (see Figure 1). In the first assessment (t0-assessment), participants fill out a retrospective screening of premenstrual symptoms [50] according to the DSM-5 criteria [7]. If participants fulfil the basic inclusion criteria and primary DSM-5 criteria (that is, five premenstrual symptoms including at least one affective symptom), a structured clinical interview regarding comorbid disorders is conducted via telephone. Afterwards, principally eligible participants are invited to complete a daily symptom rating during two consecutive menstrual cycles, as requested by the DSM-5 and ACOG criteria. During one of the premenstrual (luteal) phases within these two cycles, the participants fill in the pre-treatment assessment (t1-assessment) including primary (impairment of premenstrual symptoms) and secondary outcome measures (coping with premenstrual symptoms, social support, quality of partnership, level of stress). If the participants fulfil the diagnostic criteria of a severe PMS or PMDD with regard to the daily symptom rating, they are randomly assigned to either the treatment or the wait-list control group. In addition, they are randomized to one of two psychologists who will be the responsible psychologist throughout the study.

Participants randomized to the treatment group receive the *Praemensis* Programme, a CBT-oriented self-help treatment lasting 8 weeks. After completion of the treatment, participants complete the daily symptom rating and the questionnaires during the luteal phase (t2-assessment).

Participants assigned to the wait-list have to wait for 8 weeks and do not receive any treatment material. After the waiting period they complete the symptom diary and the questionnaires during their luteal phase (t2-assessment). Subsequently they receive the same treatment and the same post-assessment (t3-assessment). Six months after the end of the treatment the follow-up t4-assessment is administered in the luteal phase. It consists of the same questionnaires as administered at t1, t2 and t3.

### Intervention

The treatment *Praemensis* Programme is based on CBT principles. The treatment lasts 8 weeks and its ingredients are distributed across 14 separate chapters, called modules. Before starting the treatment, participants receive detailed instructions on how to use the information, how every module is structured, on the importance of exercises and on how to deal with technical obstacles.

The treatment starts with an introduction module and ends with a module about relapse-prevention. The modules in between are divided into two arms: cognitive strategies and suggestions for behavioural changes in lifestyle. For an overview see Table 1. Consequently, except for the first and the last week, participants work on two modules in parallel. For these modules, an approximate working time of 5 hours per week is suggested.

**Table 1:** **Content of the different modules**

Week	Content of the module	
	Cognitive strategies	Behavioural changes in lifestyle
1	**Module 1:** Psychoeducation about PMS/PMDD and its aetiology	
2	**Module C1:** Psychoeducation about the role of thoughts and the relationship to feelings and behaviour (cognitive triad)	**Module B1:** Psychoeducation about the interrelation between stress and PMS and learning of relaxation techniques
3	**Module C2:** Transfer to PMS-specific context	**Module B2:** Psychoeducation about the interdependency between alimentation, sport and PMS
4	**Module C3:** Restructuring of dysfunctional cognitions	**Module B3:** Integration of sports into daily life by motivational plans and strategies
5	**Module C4:** Psychoeducation about PMS-specific myths and application of learned cognitive strategies	**Module B4:** Balanced diet and implementation into daily life
6	**Module C5:** Psychoeducation about helpful thoughts and development of new appraisals	**Module B5:** Psychoeducation about the influence of stress-related errors in reasoning
7	**Module C6:** PMS-specific behaviour (healthcare utilisation, protection, communication)	**Module B6:** Training of consumption and implementation of positive activities into daily life
8	**Module 8:** Review of the training and relapse-prevention	

PMDD, premenstrual dysphoric disorder; PMS, premenstrual syndrome.

The first module includes psychoeducation about PMS/PMDD, its aetiology, and treatment. The cognitive modules provide information about, and strategies for, identifying and modifying dysfunctional cognitions – especially PMS-specific cognitions – and PMS-specific myths and behaviours. The modules including suggestions for lifestyle changes cover topics such as stress reduction, sports, and balanced diet. In the last module, a summary as well as a plan to maintain gains and prevent relapse is provided. All modules are available for download in pdf format. They all include practical exercises for applying and practicing the provided theoretical contents. Additional audio files are provided in order to facilitate conducting these exercises, such as a tutorial about progressive muscle relaxation or an audio instruction for an exercise to collect PMS-associated thoughts.

In addition to written information and audio files, participants receive email feedback from a psychologist on a weekly basis. The psychologists are Master level clinical psychologists who are at an advanced stage of CBT training. To ensure therapist adherence to the manual they receive regular supervision by a licensed CBT therapist.

The modules and audio files, as well as the secured messaging system to communicate with the clinician, are delivered via a website.

To investigate patient compliance with the treatment, weekly email feedback on the treatment material is required. If participants do not send their feedback they receive two email reminders. If they do not reply to these reminders they are called by their therapist.

### Assessments

Clinical primary and secondary outcomes will be assessed by both self-report questionnaires and clinical interviews. To ensure compliance with the assessment, participants receive two reminders via email for every questionnaire they do not complete. If they do not reply, the interviewer or the therapist calls the participant.

#### Diagnostic assessment

Premenstrual symptoms are assessed with two instruments (screening and diary). The German DSM-IV-TR-based Questionnaire for the Screening of Premenstrual Symptoms was used for the screening as well as for the self-development of the diary [50]. It measures the different dimensions of the premenstrual symptoms in a very detailed way. The questionnaire consists of 27 items which cover the diagnostic criteria of the DSM-IV-TR and which is adapted according to the DSM-5. Three additional items measure the impairment caused by the checked symptoms (for example, "My social activities are impaired by these symptoms"). All 30 items are rated on a four-point rating scale ranging from 0 (not true at all) to 3 (absolutely true).

These 30 items are assessed first in the retrospective screening (t0). Here, the items are answered for the luteal and the follicular phase separately. Second, in the prospective diary, participants rate the 30 items on a daily basis. A total score as well as a distress index are calculated. The distress index reflects the intensity of the symptoms calculated averaged across 5 days.

#### Demographic variables

The following demographic variables are measured at t0: age, family status, sexual orientation, citizenship, country of birth, growing up country (of the participant, her mother and her father), religion, mother tongue, highest degree, employment status, height, weight, and number of births and aborts.

#### Primary outcome

**Impairment caused by premenstrual symptoms** The impairment caused by the premenstrual symptoms is measured with a self-developed questionnaire called the Premenstrual Syndrome Impairment Measure (PMS-B), based on the German Pain Coping Questionnaire [51], the Pain Disability Index [52] and the Self-Report Measure for the Assessment of Emotion Regulation Skills [53]. It is currently being validated in a pilot study. It consists of two subscales (mental and functional impairment), with seven items for each. The five-point Likert scale ranges from 0 (not true at all) to 4 (absolutely true). Mean values for the subscales as well as for the whole test are calculated.

#### Secondary outcome

**Coping with premenstrual symptoms** Coping with premenstrual symptoms is assessed by a self-developed questionnaire, the Premenstrual Syndrome Coping Measure (PMS-C). It is based on the German Pain Coping Questionnaire [51], the German Version of the Coping Strategies Questionnaire [54], the Self-Report Measure for the Assessment of Emotion Regulation Skills [53], and the Brief COPE [55]. It consists of five scales with four items each, which are replied to on a five-point scale ranging from 0 (not true at all) to 4 (absolutely true). Mean values are calculated for the subscales as well as for the total scale. This questionnaire is also being validated in a pilot study.

**Social support** The Short-Form Social Support Questionnaire [56] is employed to measure the availability of social support. This 22-item measure consists of three subscales: emotional support, practical support, and social integration. Four additional items measure satisfaction with the social support and availability of a person of trust. Each item is rated on a five-point scale ranging from 1 (not true at all) to 5 (absolutely true). A mean value for the whole scale is calculated [57]. A moderate to high reliability has been found in different studies [57, 58]. A good to very good factorial validity has been demonstrated [57].

**Quality of partnership** The quality of partnership is measured with a 25-item measure, the Questionnaire for the Assessment of Relationship Quality [59]. It consists of six scales: fascination (three items), engagement for the relationship (five items), sexuality in the relationship (five items), perspective of the relationship regarding the future (five items), mistrust to the partner (three items), and restriction of the independency (five items). The single subscales as well as the total score can be interpreted. Good psychometric properties have been shown with Cronbach’s alpha for the subscales ranging between 0.75 and 0.94 [59]. Good to very good convergent validity was demonstrated (r = 0.59 to 0.84) by two questionnaires measuring partnership quality and partnership satisfaction and a further questionnaire measuring depressive mood [59].

**Level of stress** The Perceived Stress Scale is used to assess the appraisal of situations in life as stressful, particularly as "unpredictable, uncontrollable, and overloaded" [60]. It consists of ten items forming the aggregate value. The items are rated on a five-point scale from 0 (never) to 4 (very often). Good reliability (α = 0.89) as well as good convergence (r = 0.73) with the State-Trait Anxiety Inventory and good divergent validity (r = 0.02 to 0.18), for example with the Sensation Seeking Scale, are supported in a recent study [61].

### Statistical analysis

All analyses of the outcomes will be conducted as intention-to-treat analyses. Between-group changes at post-treatment on the impairment and the use of coping strategies will be calculated by using a 2 (pre-treatment/post-treatment) × 2 (experiment group/waiting list) MANOVA. *Post hoc* comparisons will be applied if clarification of the main effects of the MANOVA is needed. Effect sizes between the groups will be calculated with Hedges’ g.

## Discussion

The current study evaluates an iCBT self-help for PMS, the *Praemensis* Programme, and aims to reduce the high impairment caused by the symptoms and improve the coping abilities. Benefits and limitations of this approach are discussed below.

We developed a standardised, manualized intervention which all participants have to pass through. However, depending on the severity, the women differ in their need for treatment and in which modules are appropriate for them [62]. This heterogeneity in treatment-related needs might be especially relevant in light of the high variety of PMS-related symptoms [40]. Nevertheless, to our knowledge, a standardised manualized approach for treating PMS does not yet exist. Consequently, our primary aim and the first step is now to evaluate this standardized and manualized intervention before adapting and individualising it. Furthermore, individually tailored interventions are difficult to research in RCTs [40]. A higher flexibility in selecting the modules should be tested in further studies. First studies show promising results for a tailored iCBT [63].

To assess the impairment and coping strategies we developed two questionnaires. To the best of our knowledge, German questionnaires which assess the impairment by and coping with PMS have not yet been developed. Since we wanted to make sure that we were specifically measuring PMS-related impairment and coping, instead of impairment and coping in general, we decided to use the newly developed questionnaires.

The careful methodical design of the study allows for a better understanding of the contradictory findings about CBT for PMS [39]. First, it is an RCT, whereas many previous studies did not even include a control group [64]. Second, we calculate the point of time of the luteal phase individually for every woman. This procedure enables us to assess the primary and secondary outcome exactly within the luteal phase. Third, the participants are diagnosed carefully including a structured clinical interview for comorbid disorders, a retrospective symptom rating and a symptom diary during two cycles. The complex diagnostics, in particular the diary (which requires a high motivation), is a particularity of the current trial. Although the daily symptom rating represents one of the diagnostic criteria in the DSM-5 [7] it is often neglected in other studies as well as in clinical routine [10, 40, 62]. The new criteria of the DSM-5 have not yet been proven in practice. Thus, our study might help to test their practicability.

It has been argued that the diagnosis PMDD can lead to a pathologization of the menstrual cycle [8, 65]. The programme addresses this problem. First, detailed diagnostics help to prevent pathologization [8] by differentiating between women who experience common cycle changes and women who experience considerable impairment needing treatment [8, 62]. Second, the programme is tailored exclusively to clinically impaired women. Third, the intervention takes women’s premenstrual experiences into account without labelling every premenstrual change as pathologic [66, 67] and without labelling the experiences as fixed categories caused by hormonal or neurotransmitter imbalance [68]. This is realised by considering different theoretical models (see [38, 69, 70]) and hence incorporating the role of social and environmental factors in the psychoeducational part as well as when suggesting appraisal and coping strategies.

The counterpart of a pathologization is to deny premenstrual symptoms [71] which might also be problematic as many women suffering from PMS do not feel that they are being taken seriously [41, 70]. Validation of their feelings and support for their distress is one of the most important aspects of a psychological or medical treatment [41, 72]. In our study, the prospective diary avoids a denial of premenstrual changes by linking the symptoms clearly to the menstrual cycle. In addition, within the framework of the intervention, the women are validated and taken seriously in their feelings and cognitions.

Future studies should focus on comparing the *Praemensis* Programme with a different psychological treatment as a control condition [38]. Technical developments such as using apps for the diary could be useful and should be evaluated as well. The implementation of internet-based interventions in the healthcare system continues to be difficult; for instance, due to a lack of uptake and engagement [73]. Nevertheless, first attempts at an implementation are taking place in the United Kingdom [42, 73]. If proven effective, an implementation of the *Praemensis* Programme in the healthcare system would be desirable in the long term and should be pursued.

The *Praemensis* Programme has at least three important implications. First, it addresses the problem of insufficient care of women suffering from severe PMS or PMDD by providing treatment. Second, a careful evaluation of a CBT treatment for PMS will contribute to the question of whether CBT is promising for the treatment of PMS/PMDD and might thus shed some light on contradictory findings regarding the efficacy of CBT for PMS [39]. Third, it offers treatment for a greater number of women due to its internet-based design. To our knowledge, this study is the first study investigating internet-based therapy for PMS.

## Trial status

Participant recruitment began on 1 July 2013 and is still ongoing.

## Abbreviations

ACOG: American Congress of Obstetricians and Gynecologists
CBT: cognitive behavioural therapy
GnRH: gonadotrophin-releasing hormone
iCBT: internet-based cognitive behavioural therapy
MANOVA: multivariate analysis of variance
PMDD: premenstrual dysphoric disorder
PMS: premenstrual syndrome
RCOG: Royal College of Obstetricians and Gynaecologists
RCT: randomised controlled trial
SSRI: selective serotonin re-uptake inhibitor.
